# Long lived photogenerated charge carriers in few-layer transition metal dichalcogenides obtained from liquid phase exfoliation†

**DOI:** 10.1039/d3na00862b

**Published:** 2023-11-29

**Authors:** Floriana Morabito, Kevin Synnatschke, Jake Dudley Mehew, Sebin Varghese, Charles James Sayers, Giulia Folpini, Annamaria Petrozza, Giulio Cerullo, Klaas-Jan Tielrooij, Jonathan Coleman, Valeria Nicolosi, Christoph Gadermaier

**Affiliations:** a Area Science Park Basovizza S.S. 14 Km 163.5 34149 Trieste Italy floriana.morabito@areasciencepark.it; b Dipartimento di Fisica, Politecnico di Milano Piazza L. da Vinci 32 20133 Milano Italy christoph.gadermaier@polimi.it; c Center for Nano Science and Technology @PoliMi, Istituto Italiano di Tecnologia Via Rubattino 81 20134 Milan Italy; d CNR-IOM, Consiglio Nazionale delle Ricerche Istituto Officina dei Materiali Trieste Italy; e School of Physics, CRANN & AMBER Research Centres, Trinity College Dublin Dublin D02 Ireland; f Catalan Institute of Nanoscience and Nanotechnology ICN2 UAB Campus Bellaterra (Barcelona) 08193 Spain; g TU Eindhoven, Department of Applied Physics Den Dolech 2 5612 AZ Eindhoven The Netherlands

## Abstract

Semiconducting transition metal dichalcogenides are important optoelectronic materials thanks to their intense light–matter interaction and wide selection of fabrication techniques, with potential applications in light harvesting and sensing. Crucially, these applications depend on the lifetimes and recombination dynamics of photogenerated charge carriers, which have primarily been studied in monolayers obtained from labour-intensive mechanical exfoliation or costly chemical vapour deposition. On the other hand, liquid phase exfoliation presents a high throughput and cost-effective method to produce dispersions of mono- and few-layer nanosheets. This approach allows for easy scalability and enables the subsequent processing and formation of macroscopic films directly from the liquid phase. Here, we use transient absorption spectroscopy and spatiotemporally resolved pump–probe microscopy to study the charge carrier dynamics in tiled nanosheet films of MoS_2_ and WS_2_ deposited from the liquid phase using an adaptation of the Langmuir–Schaefer technique. We find an efficient photogeneration of charge carriers with lifetimes of several nanoseconds, which we ascribe to stabilisation at nanosheet edges. These findings provide scope for photocatalytic and photodetector applications, where long-lived charge carriers are crucial, and suggest design strategies for photovoltaic devices.

## Abstract 2

Liquid-phase exfoliation enables tiled nanosheet films of 2D semiconductors with exceptionally long carrier lifetime for light harvesting and sensing.

## Graphical abstract

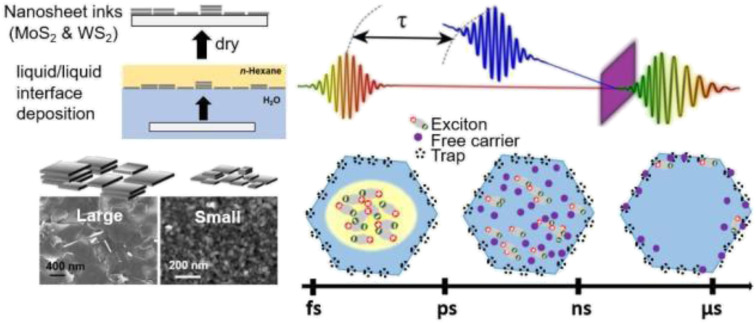

## Introduction

Light harvesting processes, such as photovoltaics and photocatalysis, convert the absorbed light energy into other forms, such as electrical or chemical energy. Likewise, light sensing relies on the generation of an electrical signal in response to light. For either of these processes to happen, light needs to be absorbed by one or more active materials, converting the photonic energy into electronic excitations of the absorber *via* the creation of electron–hole pairs (either as free carriers or bound in the form of excitons). To harness their energy or their encoded information, carriers must remain in an excited state (*e.g.* electrons in the conduction band and holes in the valence band of a semiconductor) for long enough to catalyse a chemical reaction or to be extracted at the electrodes of a photovoltaic or photodetector device.

Two-dimensional semiconductors such as layered transition metal dichalcogenides (TMDs) combine intense light–matter interaction, intricate exciton and valley physics, a wide selection of fabrication techniques, and potential for light harvesting and information processing.^1–6^ The fabrication methods for TMDs can be classified into two main categories. The first is bottom-up synthesis,^7–11^ which often necessitates expensive equipment and is primarily employed to produce monolayer films that cover a large area, ranging in the order of hundreds of microns. The second approach is top-down exfoliation, where mono- and few-layer nanosheets, typically with smaller lateral dimensions, are extracted from larger crystals or microcrystallites.^12^ Among the latter, liquid phase exfoliation (LPE) stands out as it can be applied to a wide range of layered materials.^13–17^ Furthermore, LPE offers scalability, making it suitable for mass production of inks that can be used to print TMD films onto various substrates. However, compared to monolayers,^18,19^ in TMD nanosheets obtained using LPE the non-equilibrium dynamics of photogenerated excitons and charge carriers, which is the foundation of light harvesting and sensing, is much less comprehensively studied and understood.

Here, we study the room temperature dynamics of photogenerated charge carriers in films of few-layer MoS_2_ and WS_2_ nanosheets obtained by LPE. To achieve the high film quality required for optical spectroscopy we adapt the Langmuir–Schaefer deposition technique^20^ for fabrication of tiled nanosheet films on a liquid/liquid interface, which are then transferred onto fused silica substrates. For this purpose, inks comprising nanosheets with varying average sizes and thicknesses have been employed. We investigate the charge dynamics over a temporal window from 1 ns to 100 ns using broad band transient absorption (TA) spectroscopy and the charge diffusion from 300 fs to 13 ns using spatiotemporal pump–probe microscopy. Combining these two techniques, we cover a time scale spanning six orders of magnitude – from hot carrier diffusion to the recombination of long-lived carriers – and combine spectral selectivity with the ability to disentangle the decay and diffusion of excited species. We find charge carrier lifetimes of several nanoseconds, which we attribute to stabilisation at the nanosheet edges. These lifetimes are orders of magnitudes longer than in TMDs obtained with other fabrication methods,^21–23^ which is very promising for photocatalysis and photodetector applications and suggest design strategies for photovoltaic devices.

## Results and discussion

**Fig. 1:**
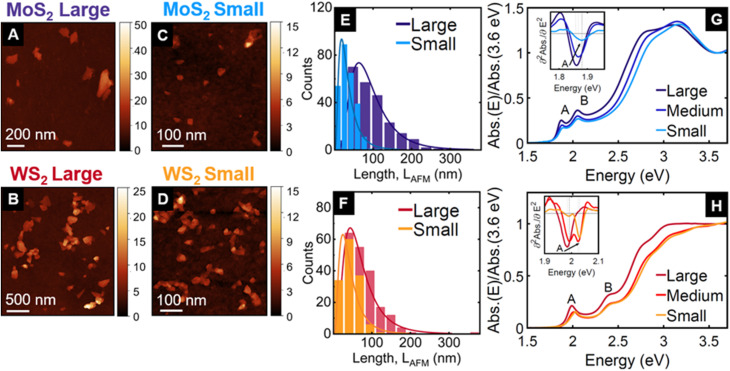
Microscopic and optical characterisation of the three size-selected fractions of MoS_2_ and WS_2_ obtained by liquid cascade centrifugation (see Methods section), after exfoliation in aqueous solution containing sodium cholate as a stabiliser. Each fraction is defined by two centrifugal acceleration values expressed in “*g*” units, where “*g*” is the Earth gravitational acceleration constant. The large fraction is stably dispersed at 1k *g* and precipitates at 5k *g*. The analogous boundary values are 5k *g* and 10k *g* for the medium, and 10k *g* and 30k *g* for the small fractions. AFM images of drop-cast nanosheets: (A) for MoS_2_ large, (B) for WS_2_ large, (C) for MoS_2_ small, and (D) for WS_2_ small. The colour encodes the height in nm. Histograms of the nanosheet lateral size distribution for large (dark blue) and small (light blue) fractions of MoS_2_ in (E), and for large (dark red) and small (orange) fractions of WS_2_ in (F). Absorbance spectra of the three fractions dispersed in aqueous sodium cholate solution normalised at 3.6 eV (large in dark blue, medium in blue, and small in light blue) of MoS_2_ in (G), and for WS_2_ (large in dark red, medium in red, and small in orange colours) in (H). The insets in (G) and (H) show the second derivative around the A-exciton, computed from smoothed absorbance spectra, for more precise determination of the peak position: 1.86 eV (1.98 eV) for MoS_2_ (WS_2_) large; 1.87 eV (1.99 eV and 2.00 eV) for MoS_2_ (WS_2_) medium; and 1.88 eV (2.03 eV) for MoS_2_ (WS_2_) small. Notice that the second derivative of WS_2_ medium fraction shows two peaks, corresponding to well resolved contributions from thicker and thinner nanosheets, respectively. Large, medium, and small fractions are the sediments collected after centrifugation between two boundaries of gravitational acceleration (*i.e.* 1–5k *g*, 5–10k *g*, and 10–30k *g* respectively). Each of those fractions correspond to a distribution of flakes with thicknesses and lateral sizes as reported in Fig. S1 and S2 (see ESI† for further details on the distributions).

We prepared dispersions of MoS_2_ and WS_2_ nanosheets from powders in aqueous solutions of the surfactant sodium cholate using sonication assisted LPE. From the resulting stock dispersion, we isolated three different fractions by liquid cascade centrifugation,^17,24^ corresponding to centripetal accelerations of 1–5k *g*, 5–10k *g* and 10–30k *g*, as described in the Methods section. Each of these fractions, named small, medium, and large, contains a different distribution of nanosheet thicknesses (*i.e.* number of layers, *N*) and lateral sizes, *L* that are characterised using statistical atomic force microscopy^20^ as depicted in Fig. 1A–F, S1, and S2† and summarised in Table S1 (see ESI†). For both MoS_2_ and WS_2_, the three fractions show different mean <*N*> and <*L*> values ranging from 2.2 to 4.4 layers and spanning from 35 to 97 nm, respectively. Note that the larger nanosheets are also thicker^24,25^ and show a distribution with longer tails towards higher *N* and *L* values, which is a typical observation for nanosheets isolated through liquid cascade centrifugation.^26–28^

The absorbance spectra of the three fractions in liquid dispersion, depicted in Fig. 1G and H, show the well-known A and B excitonic resonances.^29,30^ These excitons are associated with direct transitions between valence band maxima and conduction band minima at the *K* point, where spin–orbit interaction leads to a splitting in the valence band of a few 100 meV.^31^ The A-exciton peak energy, which is more precisely determined from the second derivative of the absorbance (Fig. 1G and H, inset), shows the expected blueshift in thinner nanosheets^24,25^ (from 1.87 to 1.89 eV for MoS_2_, and from 1.98 to 2.03 eV for WS_2_ fractions). While all *N* and *L* distributions show only one peak, the medium WS_2_ fraction shows a double peak in the absorbance 2nd derivative, indicating two dominant *N* values. This highlights an important difference in characterising the dispersions with microscopy after deposition or by their absorbance spectra in solution. The histograms in Fig. 1E, F, S1, and S2† represent the number of nanosheets that fall into a certain size range. In the absorbance spectrum, the contribution of each nanosheet increases with increasing area (approximately *L*^2^) and increasing *N*. As a consequence, the larger and thicker nanosheets are represented more prominently in the absorbance spectra. Hence, if the absorption peaks are sufficiently narrow, a longer tail of the *N* distribution towards larger values can yield a second maximum in the absorbance.

We prepared tiled nanosheet films from the three fractions of MoS_2_ and WS_2_, respectively, *via* a customised Langmuir–Schaefer deposition^20^ (after removing surfactant residues and changing the solvent to isopropyl alcohol (IPA), see Methods section) from a water–hexane interface onto fused silica substrates. In this process, the nanosheets of few-layer thickness obtained from LPE arrange in a mosaic with close lateral packing, but with minimal vertical stacking (see SEM images in Fig. S3†) and excellent optical quality for time-domain spectroscopic investigation.

**Fig. 2:**
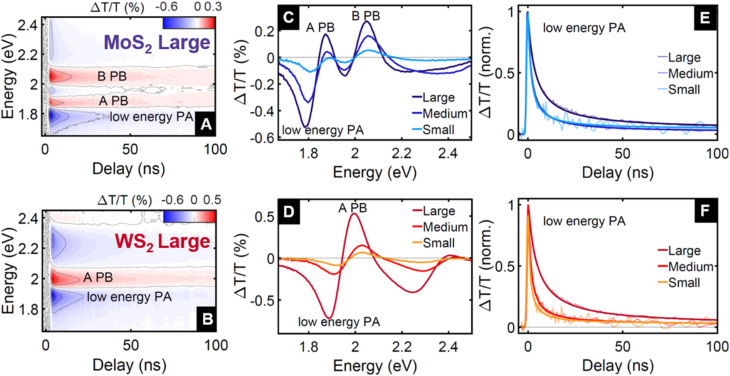
TA maps of (A) MoS_2_ and (B) WS_2_ thin films from the large (*i.e.* flakes distribution of thicknesses up to 12 layers, and lateral sizes up to 225 nm) fraction up to 100 ns delay. The map shows the differential transmission Δ*T*/*T* as a function of probe energy and relative delay between pump and probe pulses. The pump photon energy was 3.49 eV. Experiments were performed in air at room temperature. The colourmap is used to easily identify an increase of transmission (photobleaching, PB) in red and a decrease of transmission (photoinduced absorption, PA) in blue. The main features observed are the two PB peaks around the A and B exciton resonances, and the PA at low energy, which is a signature of charge carriers. Spectra are cuts of the TA map for MoS_2_ in (C) and WS_2_ in (D) at a fixed delay of 1 ns. Normalised dynamics are cuts at fixed probe energies; 1.8 eV for MoS_2_ in (E) and 1.9 eV for WS_2_ in (F). Transparent lines are experimental data, non-transparent lines are fits using eqn (1).

We investigated the dynamics of photogenerated charges on time scales up to 100 ns using TA spectroscopy with pump pulses of 3.49 eV (355 nm) and approximately 1 ns duration, and broadband probe pulses covering the spectral range 1.65–2.58 eV (480–750 nm). The TA maps for the fractions MoS_2_ and WS_2_ Large in Fig. 2A and B show the relative differential transmission Δ*T*/*T* as a function of both probe energy and delay time between the pump and probe pulses. They exhibit the typical photobleaching (PB) features of the A- and B-exciton resonances (labelled A PB and B PB), accompanied by a series of photoinduced absorption (PA) peaks (the most dominant of which is labelled low energy PA). The decreased absorption observed as A PB and B PB has been attributed to the depopulation of the valence band and Pauli blocking from population in the conduction band.^32–37^ Excited state population also enables or enhances transitions towards higher excited states, seen as PA. Contrary to monolayer TMDs, where the TA signal is dominated by excitons,^18,19^ in few-layer TMDs the low energy PA originates from transitions from photogenerated charges towards trions,^32–37^*i.e.* excitons bound to a charge.^38^ Hence, we associate the temporal evolution of the low energy PA with the dynamics of these photogenerated charges.

The transient response, reported in Fig. 2A and B, shows similar PB and PA features for all investigated fractions of MoS_2_ and WS_2_. We find that the transient spectra, shown in Fig. 2C and D, are blue-shifted by a few 10 meV for thinner nanosheets, which is fully consistent with the absorbance spectra discussed previously. The spectral shape does not vary appreciably with pump–probe delay (see Fig. S4†), indicating that photogenerated charge carriers dominate not only the low energy PA feature, but also the other PA and PB features.

The dynamics of the low energy PA reported in Fig. 2E and F reveal that the charge populations in both MoS_2_ and WS_2_ exhibit a significantly slower decay in the largest nanosheet fraction. The dynamics show a non-exponential decay and they can be accurately described by a model consisting of an instrument-limited rise followed by a second order power law decay (see eqn (1) below). This is typical for processes such as non-geminate recombination^39^ and will be discussed in detail below. Notably, the recombination rate tends to be slower for larger flakes.

**Fig. 3:**
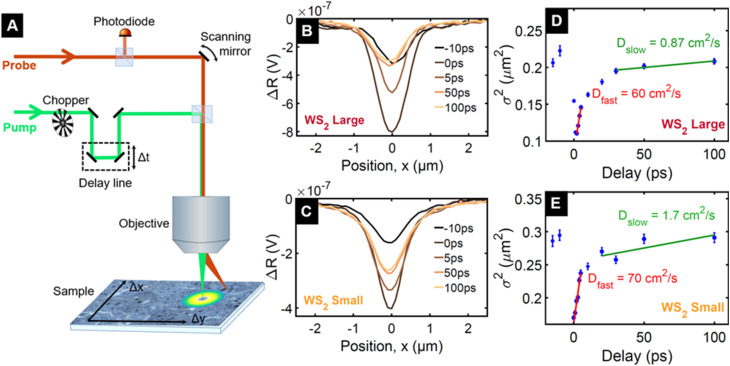
Spatiotemporal pump–probe microscopy. (A) Sketch of the setup used to track charge carrier diffusion. (B) and (C) Temporal evolution of spatiotemporal profiles reported at five selected delays (*i.e.* −10 ps, 0 ps, 5 ps, 50 ps and 100 ps) for WS_2_ large (*i.e.* flakes distribution of thicknesses up to 12 layers, and lateral sizes up to 225 nm) and WS_2_ small (*i.e.* flakes distribution of thicknesses up to 7 layers, and lateral sizes up to 110 nm), respectively. Since pump and probe pulses have a repetition period of 13 ns, the spatial profile at negative delay is due to excitation by the preceding pump pulse and thus corresponds to a pump–probe delay of 13 ns. (D) and (E) Spatiotemporal profiles expressed as the square width (*σ*^2^) of a Gaussian fit to the spatial intensity distribution for WS_2_ large and WS_2_ small, respectively. The error bars represent the standard deviation from the fitting routine. The difference in width at time zero is likely due to the slightly different focussing conditions in the two measurements. Different focuses will have different pump spot sizes and therefore dictate the spatial extent of the initial electronic excitation.

In order to understand the origin of the remarkably slow dynamics, we measure the diffusivity using spatiotemporal pump–probe microscopy^40^ on the fractions named WS_2_ small and WS_2_ large. This allows us to determine if the dynamics are associated with charges or with phonon heat, as the electron system has a much larger diffusivity than phonon heat. The choice of WS_2_ is motivated by its larger TA signal, well-separated spectral features, and narrower linewidth compared to MoS_2_. The fractions at opposite ends of the size selection enable the study of the role of nanosheet size and thickness on the carrier diffusion. We use a pump photon energy of 2.41 eV (515 nm) and a probe energy of 1.99 eV (623 nm), in resonance with the A PB peak and record differential reflectivity (Δ*R*/*R*) spatial profiles at different time delays, as described in the Methods section and shown in the sketch of the experimental setup depicted in Fig. 3A. The tightly focused pump generates an excited state population with a Gaussian spatial profile of initial *σ*^2^ = 0.09 μm^2^ (*σ* = 300 nm), whose temporal evolution is shown in Fig. 3B and C. Since the excited region is much larger than the size of the individual nanosheets, hundreds of nanosheets are excited simultaneously and hence the evolution of the spatial profile can contain contributions from heat or charge diffusion both inside each nanosheet and between adjacent nanosheets.

The spreading of the spatial profile of the photoexcited region is shown in Fig. 3D and E as the time-dependence of *σ*^2^ of the Gaussian curve. We identify an initial fast spreading during the first few picoseconds and a gradual crossover to a much slower spreading, which becomes negligible after 100 ps. During the slow spreading, the spatial profile widens only modestly from *σ*^2^ = 0.20 μm^2^ to *σ*^2^ = 0.21 μm^2^ (*i.e.* from *σ* = 450 nm to *σ* = 460 nm) for WS_2_ large and from 0.26 μm^2^ to 0.29 μm^2^ (510 to 540 nm) for WS_2_ small. The fact that the slow spreading is limited to a few tens of nanometres, which is the size of the smaller nanosheets within the distribution, suggests that the underlying heat or charge diffusion is limited to the individual nanosheets.

The time-dependence of *σ*^2^ allows to determine the diffusion coefficients *D* according to Fick's law. We obtain *D* = 60 cm^2^ s^−1^ and *D* = 70 cm^2^ s^−1^, for WS_2_ large and WS_2_ small, respectively, for the initial fast diffusion and *D* = 0.87 cm^2^ s^−1^ and *D* = 1.7 cm^2^ s^−1^ for the subsequent slower diffusion These are clearly higher than the thermal diffusivity measured in freestanding multilayer TMD sheets (in the range from 0.2 to 0.6 cm^2^ s^−1^).^40^ Therefore we conclude that the observed signal is likely not associated with phonon heat, but originates from diffusion of photogenerated charges. On the other hand, the obtained diffusivities are one order of magnitude smaller than in a large WS_2_ monolayer sheet (980 cm^2^ s^−1^ for hot carriers, 11 cm^2^ s^−1^ for thermalised carriers),^41^ suggesting that the diffusion between nanosheets is negligible or at least significantly slower than diffusion within a nanosheet.

The time dependence of the diffusion coefficient suggests the following scenario for the photoexcitation dynamics. The pump pulse at 2.41 eV, several 100 meV above the A exciton resonances of MoS_2_ and WS_2_, creates a population of hot electron–hole pairs. These have a high mobility, as previously found in WS_2_ monolayers.^41^ After several picoseconds, the electrons and holes have thermalised to the conduction band minimum and valence band maximum, respectively,^19^ where their diffusion coefficient is greatly reduced. This diffusion proceeds for approximately 100 ps, after which the carriers are either trapped or cannot spread further due to the highly inefficient inter-nanosheet charge transfer.^42,43^

From the results of TA and spatiotemporal microscopy we can develop a more comprehensive scenario of the charge carrier dynamics. After thermalisation, the diffusion of carriers is confined to the single nanosheet and impedes spreading of the spatial profile after about 100 ps. On the other hand, charge recombination continues for tens of nanoseconds. This means that at nanoseconds delays, either the carriers are already paired into slowly recombining momentum indirect excitons,^44^ or at least one carrier polarity is still mobile and can recombine with opposite charges, for example in a Langevin-type mechanism if both charges are mobile,^39^ or in a Shockley–Read–Hall (SRH) mechanism if one of them is trapped.^45^

To investigate potential mechanisms for charge recombination, we take into account the following well-established observations: (i) the decay dynamics we observe are significantly slower compared to the sub-ns charge carrier lifetimes observed in samples obtained through bottom-up growth or mechanical exfoliation.^46–48^ Even in type-II heterostructures, where carrier polarities separate onto different materials, the decay times at room temperature are well below 1 ns.^23^ (ii) Slow charge recombination, as reported in the literature, is typically associated with stabilisation at defects such as sulphur vacancies,^49,50^ heteroatoms,^51,52^ molecules adsorbed on the nanosheet surface,^53^ and dangling bonds/trapped charges of the substrate.^54^ The main difference in the nanosheets from LPE is that they may have some residual surfactant to passivate some of these defects,^55^ and, being smaller, they have a much larger edge-to-basal plane ratio. Indeed, charge lifetimes of several nanoseconds have previously been observed in monolayers synthesized using a particular laser-assisted synthesis technique that yields a material with strongly red-shifted absorption and TA spectra due to internal strain.^56^ Since our spectra do not show such red-shift, we exclude strain as the main reason for the long charge lifetimes. However, a similarly long charge lifetime has been observed in exfoliated material with a less detailed size selection and consequently larger, thicker nanosheets.^35^ Combining these facts – the unusually long charge carrier recombination time, the possible stabilisation of charges at defects, the high defect concentration at edges, the particularly high edge-to basal plane ratio of our samples, and the strongly inhibited inter-nanosheet transfer of relaxed charge carriers – we ascribe the observed long charge recombination times to the stabilisation of one charge polarity at the edges, while the other is still mobile.

Since the mobile charges can recombine with any of the trapped charges of opposite polarity, we model the temporal evolution of the carrier population as a non-geminate recombination, whose rate is proportional to both the electron and hole populations, which we assume to be equal, and describe as a single time-dependent charge population *C*(*t*):1
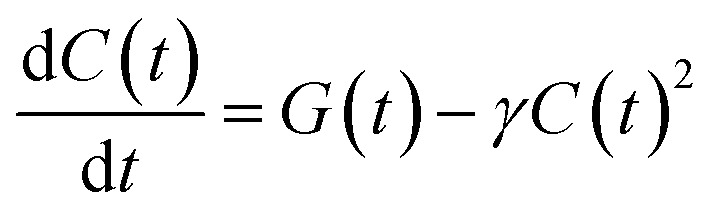
where *G*(*t*) describes charge photogeneration by the pump pulse, and *γ* is the recombination coefficient. We add a small population (a few % of the total initial population) that does not decay appreciably within the 100 ns temporal window, as it is consistent with the microsecond carrier lifetimes observed in time-resolved microwave conductivity.^57^ This simple model was used to fit the time traces in Fig. 2E and F where we find excellent agreement with the experimental data. The values obtained for the recombination coefficient *γ* decrease for increasing nanosheet size (from 2.5 × 10^−11^ cm^3^ s^−1^ to 6.6 × 10^−12^ cm^3^ s^−1^ for MoS_2_, and from 5.2 × 10^−11^ cm^3^ s^−1^ to 5.2 × 10^−12^ cm^3^ s^−1^ for WS_2_).

Compared to an exponential population decay, non-geminate charge recombination does not have fixed recombination rate *k*, but rather a rate *k*(*t*) = *γC*(*t*) that decreases with increasing pump–probe delay. Hence, rather than *τ* = 1/*k*, as is customary for an exponential decay, we define as the equivalent carrier lifetime the pump–probe delay at which the population has decreased to 1/*e* of its initial value. This value increases for increasing nanosheet size (from 4 ns to 8 ns for MoS_2_, and from 2 ns to 10 ns for WS_2_). This may seem counterintuitive, since one may expect the effect of charge stabilisation at the edges to scale with the edge-to-basal plane ratio, and hence be more pronounced for smaller nanosheets. However, as long as the edges can stabilize most of the photogenerated carriers of one polarity, any further increase in this ratio will have little impact on the carrier stabilization. On the other hand, in the larger nanosheets, the mobile charges of the other polarity on average spend more time in the basal plane and need to travel over a larger distance before recombining with an edge-stabilised countercharge, resulting in a longer charge carrier lifetime for the larger flakes.

A formalism that relates *γ* to the charge diffusion coefficient *D* is provided by Langevin recombination. However, that model assumes a uniform distribution of both charge polarities over the whole sample, while here one charge polarity is trapped at the nanosheet edges. Hence, on average the mobile charges need to travel much farther before recombining and applying the Langevin formalism to our *γ* values underestimates *D* by orders of magnitude (see ESI†). Similarly, the SRH mechanism, which explicitly considers traps, but again with a uniform distribution over the sample, does not yield a quantitative result for our situation. In summary, our model is a simplification that reproduces the temporal behaviour of the charge population very well, but the obtained *γ* values are not directly linked to the diffusion constant.

## Conclusions and outlook

We have shown long-lived photoexcited charge carriers in mosaic-like thin-films of semiconducting TMD nanosheets deposited using an adapted Langmuir–Schaefer approach, from inks obtained by sonication-assisted liquid phase exfoliation. Our samples exhibit much longer room-temperature carrier lifetimes than TMD monolayers or even heterobilayers with type-II band alignment,^21–23^ which are purpose-built for light harvesting applications. We attribute this behaviour to stabilisation of carriers at defect-rich edge regions of the nanosheets, whose lateral sizes range from tens to hundreds of nanometres. Slower recombination is observed for larger nanosheets, which also have a lower diffusion constant *D* for relaxed carriers. In a broader context, the long-lived carriers are stabilised at nanosheet edges, providing an excellent basis for photocatalytic activity of fabricated Langmuir-type thin-films.^58^ The size selection *via* liquid cascade centrifugation allows to optimise the trade-off between longer carrier lifetimes of thicker nanosheets and the more favourable ratio of edge to basal plane of smaller ones. Trapping at the edges may impede extraction of one charge polarity, which is not an issue for high efficiency photodetectors.^59^ On the other hand, photovoltaics requires extraction of both charge polarities. We envisage that the recently developed covalent interconnection of nanosheets,^42,43^ which enables efficient inter-nanosheet charge transport, will ultimately enable the fabrication of macroscopic photovoltaic cells using tiled films of 2d semiconductor as the active layer. While photocurrents have already been demonstrated,^60^ photovoltaic performance will require a more in-depth study of the ink processing^61^ and charge stabilisation^55,62,63^ to find the optimal balance between long lifetimes and high mobilities of photogenerated carriers.

## Methods

### Liquid phase exfoliation (LPE)

WS_2_ and MoS_2_ dispersions were prepared by sonicating 1.6 g material powder purchased from Sigma-Aldrich in 80 mL aqueous sodium cholate (SC) solution, (*C*_SC_ = 2 gL^−1^). The mixture was sonicated by a solid flathead tip, model Q500 from Fisher scientific, for 1 h at 60% amplitude with a pulse of 6 s on and 2 s off. Overheating was avoided using a cooling system model F32-HL from Julabo, connected to a jacketed beaker from Ace Glass Incorporated. The temperature of the cooling liquid was kept constant at 5 °C. Dispersions were centrifuged at 3000*g* (where *g* = 9.81 ms^−2^ is the Earth's gravitational acceleration) for 1.5 h in a centrifuge from Thermo scientific. After discarding the supernatant, the sediment was collected in 80 mL of fresh solvent and sonicated a second time for 7 h at 60% amplitude with a pulse of 6 s on and 2 s off. The resulting stock dispersion was subsequently fractionated by liquid cascade centrifugation.

### Liquid cascade centrifugation

Liquid cascade centrifugation is a multi-step procedure with sequentially increasing centripetal acceleration (2 h per run in a Hettich Mikro220R centrifuge equipped with a fixed-angle rotor 1195A, 1.5 mL polypropylene tubes), at constant temperature of 10 °C. Centrifugation conditions are expressed as an average centrifugal field in units of 10^3^ × g (or k *g*), where *g* is the gravitational acceleration. Four acceleration values were selected for the cascade: 1k *g*, 5k *g*, 10k *g*, and 30k *g*. For centrifugation speeds up to 30k *g*, a Hettich Mikro220R centrifuge equipped with a fixed-angle rotor (1195A) was used (1.5 mL polypropylene tubes). First, the dispersion was centrifuged at 1k *g*. The sediment contained mostly unexfoliated material and was discarded. In the next step, the supernatant was centrifuged at 5k *g*. The sediment was collected and redispersed in fresh solvent for further analysis. This first collected sediment is labelled as the fraction “1–5k *g*”, *i.e.* the fraction of TMD nanosheets that are stably dispersed at 1k *g* but precipitate at 5k *g*, to indicate consecutive centrifugation steps. The procedure was reiterated with the supernatant to produce additional fractions “5–10k *g*” and “10–30k *g*”. A solvent transfer was necessary to remove residues of the surfactant (*i.e.* sodium cholate). The dispersions were washed out in IPA before film deposition: the dispersions were centrifugated at 30k *g* per 2 h, the supernatant (*i.e.* solvent made of aqueous solution containing sodium cholate) was discarded and the collected sediment was redispersed in fresh IPA solvent to make the deposition step easier, avoiding material aggregates due to surfactant residues.

The three fractions are reported in figures using simple labels referring to the different mean nanosheet lateral size for an easier understanding: large (*i.e.* 1–5k *g*), medium (*i.e.* 5–10k *g*), and small (10–30k *g*).

### Deposition

Formation of tiled films of single-nanosheet thickness is achieved by a modified Langmuir–Schaefer approach as previously reported.^64^ A Teflon stand (10 cm long) supporting the substrate is placed in a beaker (about 100 mL) of deionised water. About 20 mL of distilled hexane is added onto the surface of the deionised water to create a liquid/liquid interface. Inks of MoS_2_ and WS_2_ nanosheets in IPA are added (∼140 μL) to the interface. The nanomaterial diffuses along the water/hexane interface and forms a thin-film as increasing amounts of material are added. This film is transferred onto a substrate, which is vertically moved through the interface. The films are left to dry in ambient air for ∼6 h. For analysis of the nanosheets by microscopy, Si/SiO_2_ substrates with 300 nm oxide thickness and root-mean-square roughness 0.1 nm were used. For the time-resolved measurements, transparent 1 mm thick fused silica was used.

### Atomic force microscopy (AFM)

For AFM measurements, a Dimension ICON3 scanning probe microscope (Bruker AXS S.A.S.) was used in ScanAsyst mode (non-contact) in air under ambient conditions using aluminium coated silicon cantilevers (OLTESPA-R3). The concentrated dispersions were diluted with IPA to optical densities <0.1 at 300 nm. A drop of the dilute dispersions (15 μL) was flash-evaporated on pre-heated (175 °C) Si/SiO_2_ wafers. After deposition, the wafers were rinsed with ∼15 mL of water and ∼15 mL of IPA and dried with compressed nitrogen. Typical image sizes ranged from 12 × 12 μm^2^ for larger nanosheets to 3 × 3 μm^2^ for small nanosheets at scan rates of 0.5–0.8 Hz with 1024 lines per image. Previously published length corrections were used to correct lateral dimensions from cantilever broadening and pixilation effects by calibration using transmission electron microscopy (TEM).^26,65^

### Scanning electron microscopy (SEM)

SEM is performed with a Carl Zeiss Ultra SEM operating at 2–5 kV with a 30 μm aperture. Images are acquired using the secondary electron detector. The sample substrate is a piece of SiO_2_/Si wafer (0.5 × 0.5 cm^2^) with an oxide layer of 300 nm.^55^

### Static absorption

The absorption spectra were measured in dispersion using a 10 mm optical length quartz cuvette in a PerkinElmer Lambda 1050 spectrophotometer, equipped with an integrating sphere, with the slit width set to a wavelength resolution of 2 nm, at a step of 1 nm.

### Transient absorption

A Pharos femtosecond laser by Light Conversion based on a diode pumped Yb: KGW crystal with fundamental wavelength at 1032 nm, pulse duration of approx. 200 fs and repetition rate of 2 kHz was used for white light generation. For this, the laser fundamental was focused into a 2 mm thick sapphire crystal, resulting in a broadband pulse with spectrum ranging between 470 nm and 700 nm, which was used as the probe. The differential transmission signal, defined as Δ*T*/*T* = (*T*_pump on_ − *T*_pump off_)/*T*_pump off_ was recorded using a fast visible camera (Stresing FLCC3001-FFT) coupled to a Princeton Instruments spectrometer as detector. The pump pulses were provided by a Q-switched Nd: YAG laser (Picolo by InnoLas), emitting pulses with a width of approx. 1 ns at 1064 nm fundamental wavelength. The third harmonic at 3.49 eV (355 nm) of these pulses is sent onto the sample with an 1/*e*^2^ diameter of 400 μm and a fluence of 140 μJcm^−2^. To synchronise the pump and probe pulses, the trigger of the Pharos laser at 2 kHz was sent to a digital delay generator (model DG 645 from Stanford Research Systems) with less than 25 ps rms jitter, and used to trigger the Picolo to control the relative delay between pump and probe pulses.

### Spatiotemporal pump–probe microscopy

We used the set-up as described in ref. 41. A mode-locked laser emits 100 fs pulses centred at 1030 nm at a repetition rate *f*_rep_ = 78 MHz. Most of the power was used to pump an optical parametric oscillator, which generates signal photons with tuneable wavelengths between 1340 and 2000 nm. The probe beam (*λ*_probe_ = 623 nm) was obtained from the third harmonic of the signal output and scanned over the sample area of interest using a 4f-imaging system equipped with a galvanometric mirror (Optics in Motion OIM 101).^41^ The pump beam was obtained from the second harmonic of the fundamental laser (*λ*_pump_ = 515 nm), and it was modulated using a mechanical chopper. The pump passed over a mechanical delay line (Newport DL225) and then it combined with the probe beam before the objective lens (NA 0.67) using a dichroic mirror. The reflected probe beam was measured using a photodetector and the pump-induced reflectivity change (Δ*R*) was obtained by demodulating at the chopper frequency using a lock in amplifier. By scanning the position of the probe beam (Δ*x*) with respect to the stationary pump, the spatial profile of the transient reflection signal, Δ*R*(Δ*x*), was measured. Obtaining these spatial profiles at different pump–probe delay times allows for the observation of diffusion.

## Conflicts of interest

The authors declare no competing conflict of interest.

## Supplementary Material

NA-006-D3NA00862B-s001
