# Supplementary material for: Long lived photogenerated charge carriers in few-layer transition metal dichalcogenides obtained from liquid phase exfoliation
Source: Nanoscale Adv. 2023 Nov 29;6(4):1074–83. doi: 10.1039/d3na00862b (PMC10863726; doi:10.1039/d3na00862b)
Supplement: NA-006-D3NA00862B-s001 [file NA-006-D3NA00862B-s001.pdf]

Electronic Supplementary Material (ESI) for Nanoscale Advances.
This journal is © The Royal Society of Chemistry 2023

Supporting Information for

Long Lived Photogenerated Charge Carriers in Few-Layer Transition Metal Dichalcogenides Obtained from Liquid Phase Exfoliation

Floriana Morabito[1,2,3,4], Kevin Synnatschke[5], Jake Dudley Mehew[6], Sebin Varghese[6], Charles James Sayers[2], Giulia Folpini[3], Annamaria Petrozza[3], Giulio Cerullo[2], Klaas-Jan Tielrooij[6,7], Jonathan Coleman[5], Valeria Nicolosi[5], Christoph Gadermaier[2,3*]*

1) *Area Science Park, Basovizza S.S. 14 Km 163.5 34149 Trieste, Italy*

2) *Dipartimento di Fisica, Politecnico di Milano, piazza L. da Vinci 32, 20133 Milano, Italy*

3) *Center for Nano Science and Technology @PoliMi, Istituto Italiano di Tecnologia, Via Rubattino 81, 20134 Milan, Italy*

4) *CNR-IOM, Consiglio Nazionale delle Ricerche Istituto Officina dei Materiali, Trieste, Italy*

5) *School of Physics, CRANN & AMBER Research Centres, Trinity College Dublin, Dublin D02, Ireland*

6) *Catalan Institute of Nanoscience and Nanotechnology ICN2 UAB Campus, Bellaterra (Barcelona) 08193, Spain*

7) *TU Eindhoven, department of Applied Physics, Den Dolech 2, 5612 AZ, Eindhoven, the Netherlands.*

***Corresponding Authors:**

floriana.morabito@areasciencepark.it

christoph.gadermaier@polimi.it

Quantification of nanosheet size via statistical Atomic Force Microscopy

After liquid cascade centrifugation, statistical AFM analysis was performed on drop-cast nanosheets of the individual fractions of both materials (i.e. MoS_2 and WS_2) to quantify nanosheet lateral size L and thickness in each obtained fraction. As reported in previous works, the measured thickness was converted into the nanosheet layer number N by step height analysis.[1-]

The results of the statistical AFM measurements for all size-fractions of MoS_2 and WS_2 are shown in figures S1 and S2. A summary of mean values is provided in Table S1. The first line in each Figure shows an overview of representative nanosheets found in the individual fractions. The second and third lines show histograms of the corresponding nanosheet length and layer number distributions in each fraction, respectively. After each step of the centrifugation cascade, a noticeable reduction in size and thickness occurs, alongside with a narrowing of the size distribution. This phenomenon is consistently observed as the centrifugal force progressively increases. [6, 7] Please note that the sample labeling corresponds to the specific centrifugation boundaries employed for the isolation of the individual fractions. For instance, the sample labeled as "1-5k *g*" was obtained by extracting the supernatant from a centrifugation step performed at 1000 *g*, followed by subsequent centrifugation at 5000 *g*. In all cases, the sediment collected after centrifugation was subjected to analysis and additional processing.

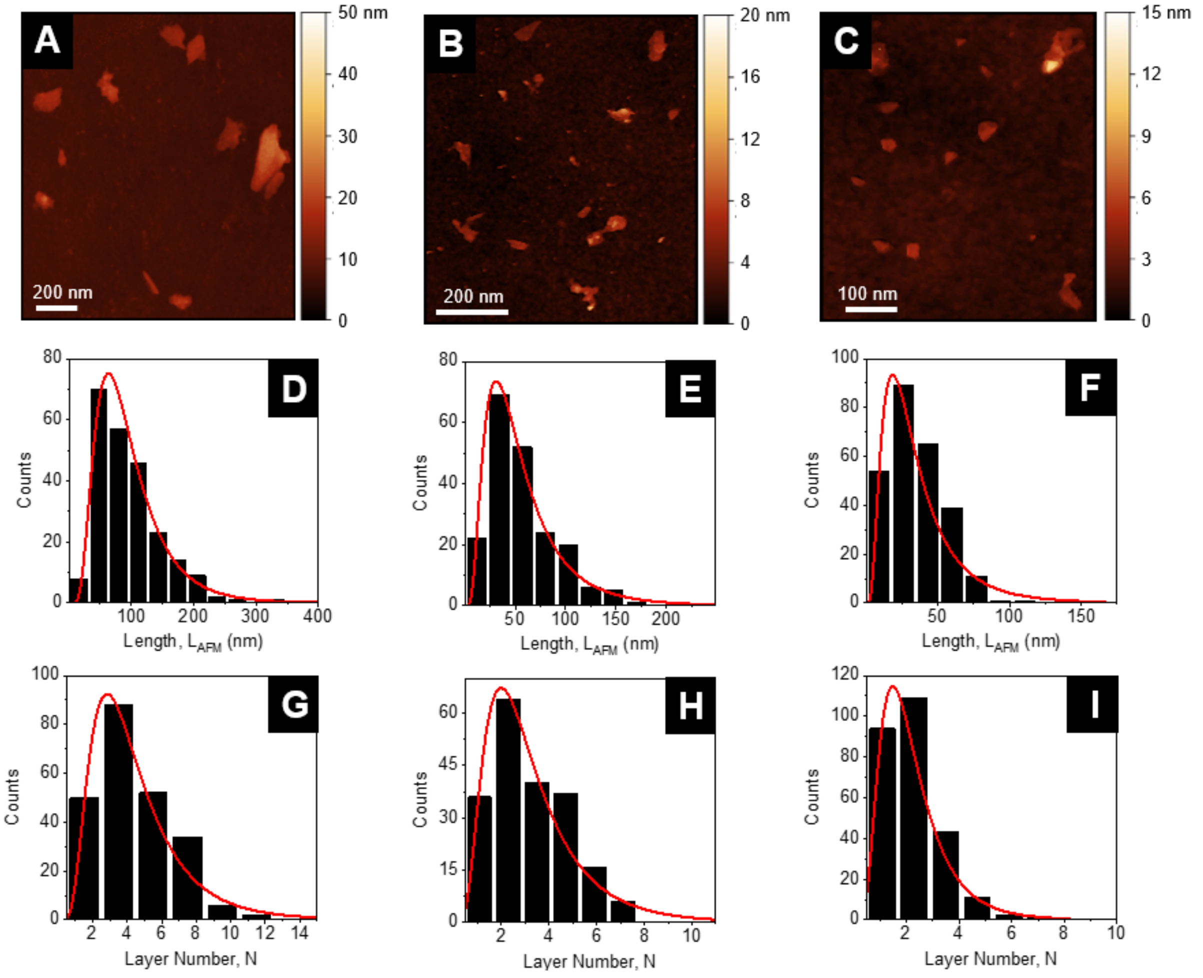

Figure S1 – AFM size and thickness distribution of MoS_2 nanosheets for all fractions obtained from centrifugation cascade. (A)-(C) Representative AFM images of the different fractions collected during the size selection. Low acceleration centrifugation boundaries 1-5k *g* in (A) for MoS_2 Large, moderate acceleration between 5-10k *g* in (B) for MoS_2 Medium, high acceleration 10-30k *g* in (C) for MoS_2 Small. (D)-(F) Histograms of the nanosheet length measured as the longest dimension of a sheet for MoS_2 Large, Medium, and Small, respectively. (G)-(I) Histograms of the nanosheet thickness converted into layer number by step height analysis, as previously reported.[8] As expected, size and thickness decrease with increasing centrifugal acceleration. After every step of the centrifugation cascade, a narrowing of the distribution is observed for both length (i.e. from (D) to (F)) and thickness (i.e. from (G) to (I)). Red lines are fits to a log-normal distribution.

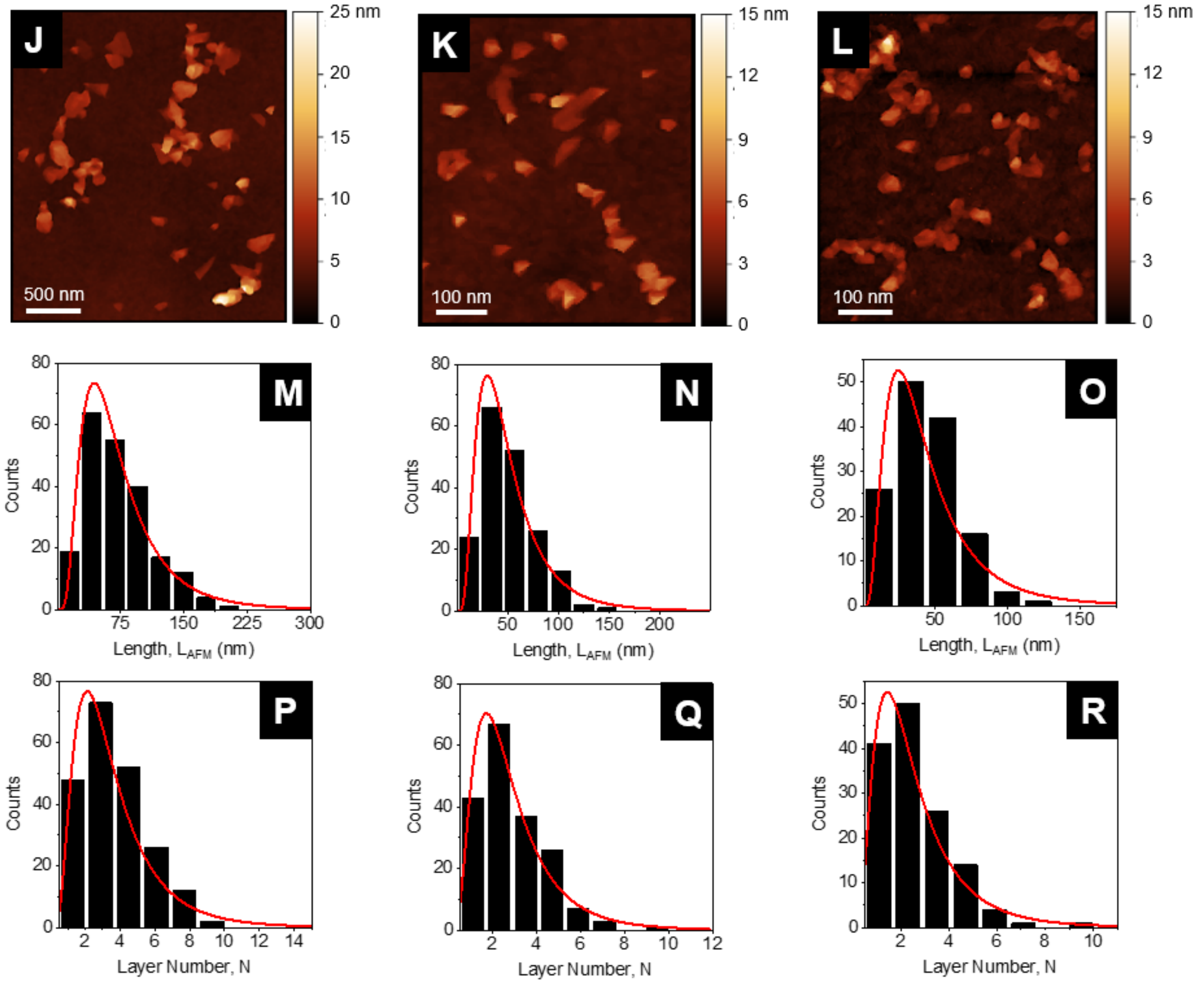

Figure S2 – AFM size and thickness distribution of WS_2 nanosheets for all fractions obtained from centrifugation cascade. (J)-(L) Representative AFM images of the different fractions collected during the size selection. Low acceleration centrifugation boundaries 1-5k *g* in (J) for WS_2 Large, moderate acceleration between 5-10k *g* in (K) for WS_2 Medium, high acceleration 10-30k *g* in (L) for WS_2 Small. (M)-(O) Histograms of the nanosheet length measured as the longest dimension of a sheet for WS_2 Large, Medium, and Small, respectively. (P)-(R) Histograms of the nanosheet thickness converted into layer number by step height analysis, as previously reported.[v] As expected, size and thickness decrease with increasing centrifugal acceleration. After every step of the centrifugation cascade, a narrowing of the distribution is observed for both length (i.e. from (M) to (O)) and thickness (i.e. from (P) to (R)). Red lines are fits to a log-normal distribution.

	<L> (nm)	<N>
MoS_2 Large	96.5 ± 3.5	4.4 ± 0.1
MoS_2 Medium	56.2 ± 2.3	3.2 ± 0.1
MoS_2 Small	35.3 ± 1.2	2.2 ± 0.1
WS_2 Large	73.5 ± 2.9	3.6 ± 0.1
WS_2 Medium	50.6 ± 1.9	2.9 ± 0.1
WS_2 Small	44.3 ± 1.9	2.6 ± 0.1

Table S1. Average nanosheet dimensions obtained via statistical atomic force microscopy (AFM) analysis. <*L*>, , and <*N*> are the arithmetic means of the nanosheet lateral size, and layer number, respectively. The error reported in the table is the standard error of the mean. The samples are labelled as "Large", "Medium", "Small" referring to the boundaries of the centripetal acceleration used during size selection via liquid cascade centrifugation (LCC) method: 1-5k g (Large), 5-10k g (Medium), 10-30k g (Small). Increasing the centripetal acceleration in steps gives access to different sediment fractions along the cascade, each of which contains nanosheets distributions centered around decreasing *N* and *L*. Note that the unit 'g' refers to the relative centrifugal acceleration expressed in multiples of the earth gravitational field.

Langmuir-Schaefer deposition: SEM images

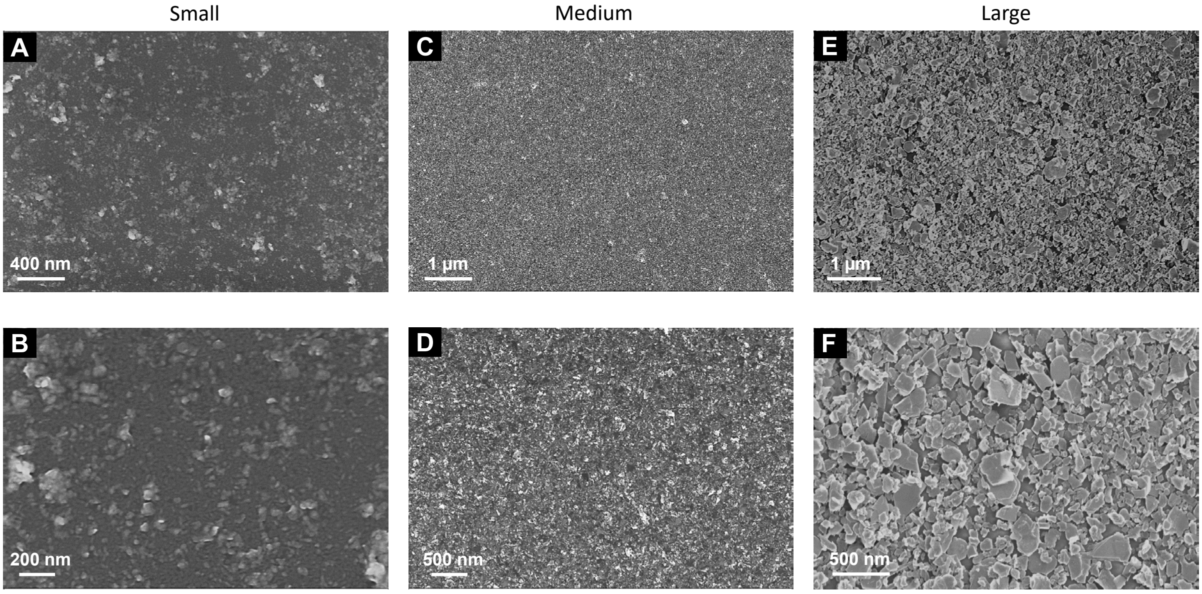

Figure S3 – SEM images of Langmuir-type deposited TMD nanosheets. Images show an overview (top row), and a zoom-in region (bottom row) of representative spots of deposited WS_2 nanosheets for the three fractions under study. (A-B) Small, (C-D) Medium, (E-F) Large WS_2 nanosheets thin-films.

Transient Absorption (TA) spectroscopy

Normalised spectra for each fraction of both materials are reported in Figure S4.

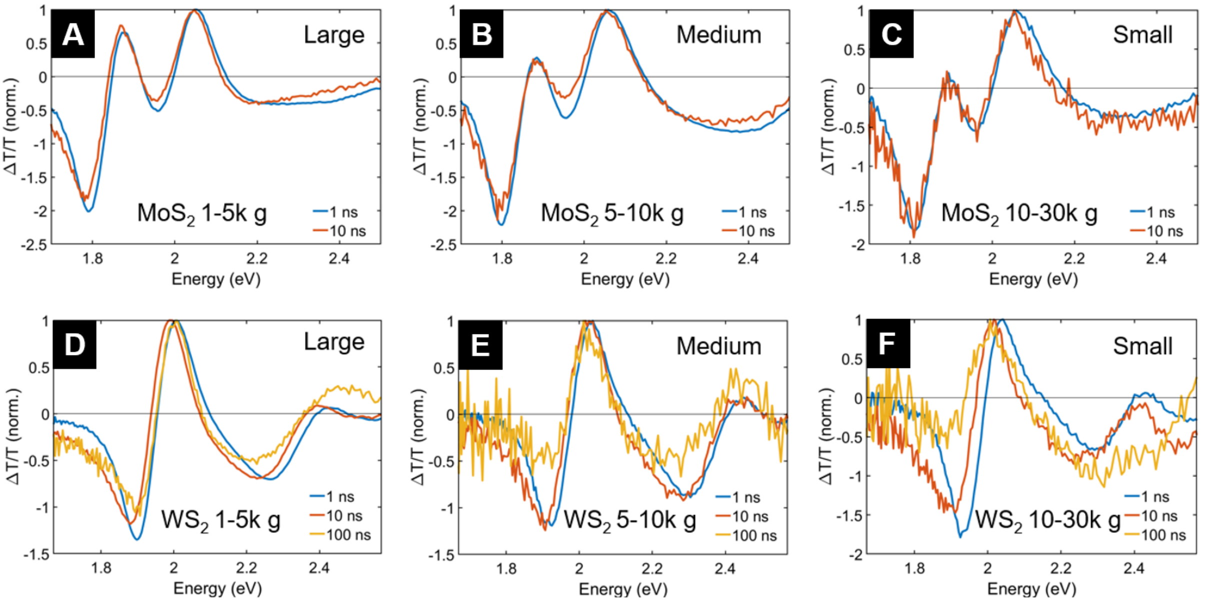

Figure S4 – Normalised TA spectra of WS_2 and MoS_2 films. (A)-(C) Normalised spectra at 1 ns and 10 ns for each fraction (i.e. Large, Medium, Small) of MoS_2. (D)-(F) Normalised spectra at 1 ns, 10 ns, and 100 ns for each fraction (i.e. Large, Medium, Small) of WS_2. In both materials, the spectral profiles do not change over time: this confirms that the TA signal is dominated by one population that we identify as charges.

References

[1] Ridings, C.; Warr, G. G.: Andersson, G. G.. Composition of the outermost layer and concentration depth profiles of ammonium nitrate ionic liquid surfaces. *Phys. Chem. Chem. Phys*. **2012**, 14, 16088-16095.

[2] Nemes-Incze, P.; Osváth, Z.; Kamarás, K.; Biró, L. P. Anomalies in thickness measurements of graphene and few layer graphite crystals by tapping mode atomic force microscopy. *Carbon* **2008**, 46, 1435-1442.

[3] Nagashio, K., Yamashita, T., Nishimura, T., Kita, K., & Toriumi, A. (2011). Electrical transport properties of graphene on SiO_2 with specific surface structures. *Journal of Applied Physics*, **110**, 024513.

[4] Backes, C.,; Higgins, T. M.; Kelly, A.; Boland, C.; Harvey, A.; Hanlon, D.; Coleman, J. N. Guidelines for Exfoliation, Characterization and Processing of Layered Materials Produced by Liquid Exfoliation. *Chem. Mater.* **2017**, 29, 1243–255.

[5] Lange, R. Z. et al. Enriching and Quantifying Porous Single Layer 2D Polymers by Exfoliation of Chemically Modified van der Waals Crystals. *Angew. Chem. Int. Ed.* **2020**, 59, 5683-5695.

[6] Backes, C. et al. Production of highly monolayer enriched dispersions of liquid-exfoliated nanosheets by liquid cascade centrifugation. *ACS Nano* **2016**, 10, 1589–1601.

[7] Ueberricke, L.; Coleman, J. N.; Backes, C. Robustness of Size Selection and Spectroscopic Size, Thickness and Monolayer Metrics of Liquid-Exfoliated WS_2. *physica status solidi (b)* **2017**, 254, 1700443.

[8] Synnatschke, K.; Shao, S.; Van Dinter, J.; Hofstetter, Y. J.; Kelly, D. J.; Grieger, S.; Haigh, S. J.; Vaynzof, Y.; Bensch, W.; Backes, C. Liquid Exfoliation of $Ni_2P_2S_6$: Structural Characterization, Size-Dependent Properties, and Degradation. *Chem. Mater.* **2019**, 31, 9127-9139.
